# Design and Application of a Core Competency Training Program for New Nurse Managers Based on the Kemp Model From Role Theory Perspective: A Pilot Study

**DOI:** 10.1155/jonm/2702060

**Published:** 2025-09-08

**Authors:** Tong Wang, Ran Zhang, Jing Li, Fei Yao, Fei Wu, Weixin Cai

**Affiliations:** Department of Nursing, Beijing Tiantan Hospital, Capital Medical University, Beijing 100070, China

**Keywords:** core competency, Kemp model, new nurse manager, role theory, role transition, training

## Abstract

**Aim:** To design nursing management training systems based on the Kemp model and role theory, enhance new nurse managers (NNMs)' core competencies, and facilitate role transition.

**Background:** Amid the diverse and intricate practical setting of policy development and execution, NNMs frequently confront numerous challenges as they transition into their roles. Although training programs strive to boost their core competencies and facilitate adaptation to new positions, most overlook the unique needs and individual traits of these managers. In addition, educational models and role theory are seldom woven into the design and assessment of such training initiatives.

**Methods:** A pre–post quasiexperimental pilot study was conducted at a tertiary hospital in Beijing, following the TREND guidelines from September to November 2024. Fourteen NNMs received core competency training program based on the Kemp model and role theory. Formative evaluation of NNMs during training used classroom questioning, homework, and reflective journal. A month post-training, summative evaluation was conducted using the Nurse Manager Competency Assessment Questionnaire and semistructured interviews. Data were analyzed using SPSS 25.0, Nvivo 12.0, and Colaizzi's thematic analysis.

**Results:** Compared with pretraining, the overall core competency (134.93 ± 11.81 vs. 146.79 ± 13.92) and behavioral competency (46.64 ± 5.47 vs. 52.93 ± 5.72) of the NNMs showed significant improvement (*p* < 0.05). Five themes emerged, including role awareness awakening and cognitive restructuring, capability adjustment in role practice, role identity and coconstruction of team culture, the tension between role expectations and career development, role cultivation and continuous professional development.

**Conclusions:** The training program, blending the Kemp model with role theory, effectively tackles the hurdles NNMs face in role transition. It boosts their core competencies and fosters role adaptation and growth, providing healthcare institutions with replicable strategies to refine support systems for nurse managers (NMs) during this phase.

**Implications for Nursing Management:** Integrating the Kemp model–role theory framework into policy-driven, competency-based leadership development enhances the role adaptability and psychological resilience of NMs across global healthcare systems.

## 1. Introduction

In the dynamic and complikex healthcare environment, new nurse managers (NNMs) shoulder the crucial responsibilities of managing nursing units or teams and directly supervising the delivery of healthcare services [1, 2]. As key members of the healthcare team, they undertake significant duties such as financial management, personnel supervision, and the implementation of healthcare policy changes [3]. Their leadership styles also influence nurses' job engagement, retention rates, and career development [4, 5]. However, they confront numerous challenges and issues [6], with the most prominent ones being high work intensity, the complexity and variability of patients' conditions, nursing staff shortages, high turnover rates, inadequate support from senior management, and limited career development opportunities [7]. In recent years, they have also had to grapple with global challenges such as population aging [8], digital health transformation [9], climate change [10], and economic pressures in healthcare [11], making the role and competency requirements for nurse managers (NMs) increasingly stringent.

Against this backdrop, nursing administrators endowed with outstanding core competencies and effective leadership skills are more likely to excel in fulfilling tasks such as ensuring high-quality nursing care, safeguarding patient safety, and retaining nursing staff [12, 13]. However, research reveals that both seasoned administrators and those newly appointed often perceive themselves as inadequately prepared [14, 15]. In particular, NNMs, who are directly promoted from clinical positions and have held managerial roles for no more than 2 years [16], are typically selected based on their clinical expertise rather than their leadership or management capabilities [17, 18]. The shift from a clinical nursing role to a nursing management role entails significant changes in the nature of work, interpersonal relationships, scope of responsibilities, and career objectives. This, in turn, leads to adjustments in the intrinsic needs of NNMs [16, 19]. Consequently, NNMs frequently encounter long-term difficulties in adapting to their roles after taking office. They may face issues such as role ambiguity and role conflicts, often feeling overwhelmed by daily management tasks, doubting their own competence, and even experiencing stress reactions like emotional exhaustion [14, 20]. These challenges impede the smooth progress of hospital management.

In recent years, a multitude of research papers have underscored the significance of increasing investment in nursing leadership development at various policy levels and across different environments. NMs require an appropriate level of education to effectively interact with other members of strategic decision-making teams [21]. In 2022, the Chinese government issued the “National Nursing Development Plan (2021–2025),” explicitly stating that during the “14th Five-Year Plan” period, it is essential to strengthen the professional training of NMs so that nursing concepts and management techniques can align with the requirements of contemporary hospital management [22]. The International Council of Nurses (ICN) [23] and the American Organization for Nursing Leadership (AONL) [24] have also pointed out that there is a continuous need to enhance the management skills and core competencies of nursing leaders to guide the clinical management practices of NMs. Research indicates that when managers find a sense of value in their roles, their job satisfaction and intention to stay increase [25]. Therefore, it is of paramount importance to provide core competency and leadership training for NNMs to help them adapt to their roles.

To facilitate the role transition and adaptation of NNMs, a series of educational and training programs have been developed, encompassing classroom instruction, experiential learning, mentorship guidance, and reflective practice [18]. AONL has also established a core competency framework to enhance the training effectiveness, work efficiency, and job satisfaction of NNMs [24]. Moreover, research has led to the development and implementation of leadership training courses tailored for NNMs, drawing on models such as the Five Forces Model [26] and the ADDLE Model [27]. However, given the increasingly complex role requirements and ever-rising expectations placed on NMs, the existing educational and training programs and courses, to a certain extent, fall short of meeting the role-related needs of NNMs.

The transition and transformation of the role for NNMs involve all-round changes in the work environment, responsibilities, and interpersonal relationships. During this critical process, clarifying one's own role positioning in the new context is the foundation for a NNM to carry out work smoothly. Role theory focuses on the roles individuals play during interactions and the laws governing these activities. Its connotations have been continuously enriched, encompassing various aspects such as role playing, role conflict, role ambiguity, role perception, role expectations, role norms, and role reconstruction [28]. It carries significant social normative implications and rich methodological value [29]. This theory is highly compatible with the role-transition phenomenon of NNMs. By leveraging role theory, we can delve deeply into the behavioral manifestations and psychological mechanisms of NNMs during the process of role transition and transformation. This enables us to guide them in correctly understanding their own roles, clarifying the expectations of all parties, and adhering to professional norms. As a result, they can complete the role transition more calmly, fostering a positive and collaborative work atmosphere and a correct value orientation within the nursing team.

**Figure 1:**
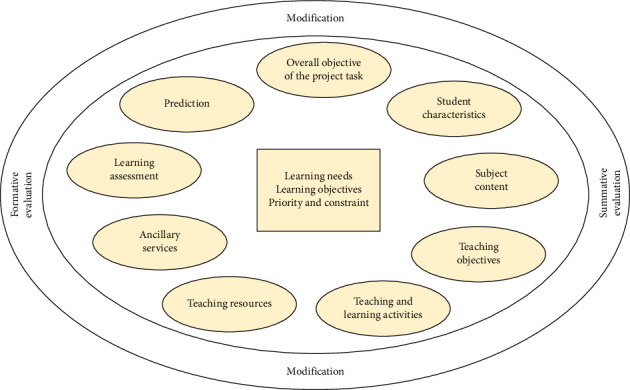
The Kemp's non-linear instructional design model. A circular framework depicting the interconnected relationships among the ten core components of the Kemp Model: ① learning needs, learning objectives, priority and constraint; ② overall objective of the project task; ③ student characteristics; ④ subject content; ⑤ teaching objectives; ⑥ teaching and learning activities; ⑦ teaching resources; ⑧ ancillary services; ⑨ learning assessment; ⑩ prediction. The absence of directional arrows emphasizes the model' s flexibility, allowing educators to begin design at any component and iterate non-linearly. The circular framework's placement of “formative evaluation,” “summative evaluation,” and “revision” highlights continuous refinement throughout the instructional process.

The Kemp model, introduced by Kemp in 1971, is a nonlinear instructional design framework [30]. It highlights four fundamental elements: teaching objectives, learner characteristics, teaching resources, and teaching evaluation. The model tackles three key issues: defining teaching objectives; analyzing learner characteristics to pinpoint the teaching starting point and, subsequently, formulating teaching strategies and methods; and implementing teaching evaluation. It also involves an appropriate arrangement of 10 instructional components (① learning needs, learning objectives, priority, and constraint; ② overall objective of the project task; ③ student characteristics; ④ subject content; ⑤ teaching objectives; ⑥ teaching and learning activities; ⑦ teaching resources; ⑧ ancillary services; ⑨ learning assessment; and ⑩ prediction). To capture the interconnected and overlapping nature of these components, Kemp eschewed a linear approach with straight lines and arrows linking teaching elements. Instead, he opted for a circular model (Figure 1) to depict this framework. The lack of directional arcs between components highlights the flexibility of instructional design, enabling educators to commence the process at any stage and proceed in any order based on practical requirements and personal teaching styles. Furthermore, the circular structure emphasizes “formative evaluation,” “summative evaluation,” and “modification,” underscoring that evaluation and refinement should be integral and ongoing throughout the entire teaching process [31].

**Figure 2:**
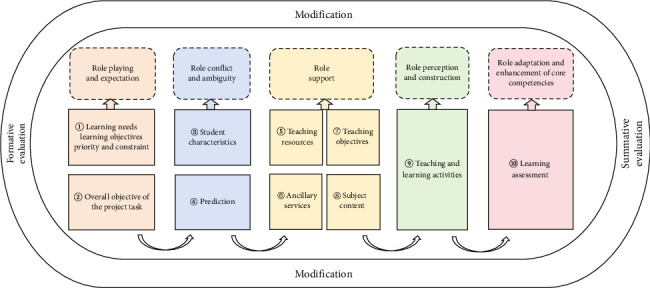
An integrated framework combining the Kemp model and role theory. A visualization of the Kemp model fused with role theory, depicting the cyclical process of new nurse managers (NNMs)' professional development. Key phases include ① Learning needs analysis: instructional designers identify obstacles and define teaching tasks; ② Role transition challenges: NNMs navigate pressures of role ambiguity and role conflict while proactively engaging in learning; ③ Holistic support system: synergy of teaching resources, ancillary services, subject content, and objectives enables effective strategy implementation; ④ Progressive role reconstruction: NNMs iteratively deepen role understanding through knowledge/experience accumulation; and ⑤ Competency evaluation: post-training assessment measures role adaptation outcomes and core competency enhancement. Central arrows emphasize the nonlinear integration of Kemp's components (e.g., learner analysis ⟶ objectives ⟶ evaluation) with role theory dynamics.

Based on the unique characteristics of role theory and Kemp model, this study took both as an integrated guiding framework to deeply explore the patterns of feature evolution of NNMs during their transition period and after training. Previously, the Kemp model has been predominantly applied in the field of education [31], including areas such as online training [32] and educational reform [33], achieving positive training outcomes. Role theory has also been widely used in pharmaceutical research and similar groups and environments [34, 35]. However, the applicability of existing research findings to the specific group of NNMs remains to be verified. In particular, the specific impacts of the combined application of the Kemp model and role theory on the core competencies and leadership abilities of NNMs are not yet clearly defined. In light of this, drawing upon the 10 instructional elements of the Kemp model, this study has made an innovative move by integrating role theory into it. The primary objective is to assess the impact of this integrated framework (Figure 2) on the development of core competence and role transition among NNMs. By doing so, we aim to furnish robust data support and a solid theoretical foundation for the establishment of a more scientific and efficient training system tailored for NNMs.

## 2. Methods

### 2.1. Research Design

A pre–post quasiexperimental pilot study was designed as a preliminary trial to help estimate the intervention effect testing the hypothesis that adopting a training program based on the Kemp model and role theory will have a positive impact on the core competencies and role adaptation of NNMs.

### 2.2. Study Population

This study was conducted in a tertiary hospital in Beijing, China. The participants were 14 NNMs who took part in a project titled “Competency Training for New Nurse Managers Based on the Kemp Model from the Perspective of Role Theory” from September to November 2024. All NNMs with less than 2 years of tenure were invited to participate in the research/training program, regardless of their age or prior professional experience. But if they were absent for ≥ 1 month for study or training during the survey period, they will be excluded from this study.

### 2.3. Sample-Size Determination

Given that this was a pilot study using a pre–post quasiexperimental design with the aim of investigating preliminary efficacy, conventional sample-size calculations, appropriate for a full-scale intervention, were not deemed necessary [36].

### 2.4. Recruitment

Recruitment will be conducted in August 2024. In the week prior to the intervention, the Principal Investigator will introduce and explain the study to the NNMs in a 2-h meeting. During this meeting, all participants will be informed about the purpose and design of the study, and they will all voluntarily agree to participate in the training program. Once the NNMs consent to participate in the study, their sociodemographic data will be collected.

### 2.5. Training Plan Design

**Figure 3:**
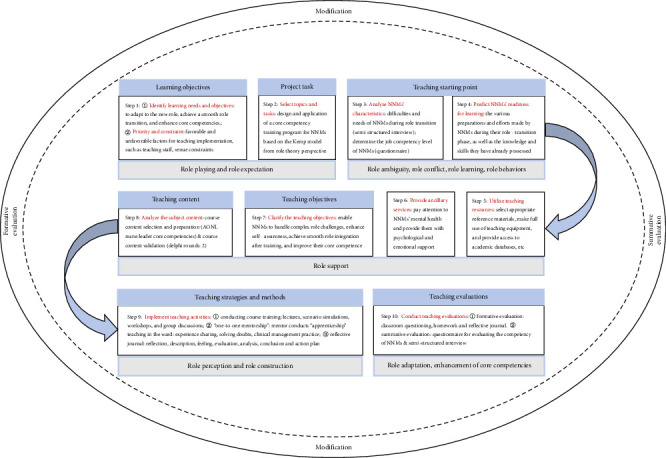
Ten-step training plan for NNMs integrating Kemp model and role theory. A sequential implementation framework that translates elements of Kemp's instructional design model and role theory into a core competency training program for NNMs that offers a comprehensive and well—organized visual presentation of key teaching elements. This implementation framework encompasses a range of crucial components, including learning objectives, the teaching starting point, teaching resources, teaching objectives, teaching content, teaching strategies, and teaching evaluation. In essence, it integrates the fundamental elements highlighted in the Kemp model—teaching objectives, learner characteristics, teaching resources, and teaching evaluation—along with the critical issues that need to be addressed. These issues involve defining clear teaching objectives; analyzing learner characteristics to pinpoint the teaching starting point, followed by the formulation of appropriate teaching strategies and methods; and finally, implementing teaching evaluation. In accordance with the research objectives and to enhance the framework's logical coherence and structural continuity, the 10 instructional components of Kemp's model were systematically reordered and restructured. This rearrangement is aimed at establishing a clear correspondence between these components, the fundamental elements of the Kemp model, and the underlying concepts of role theory. Abbreviations: NNMs, new nurse managers; AONL, American Organization for Nursing Leadership.

Qualitative (for need assessment and establishing effectiveness of the intervention based on role theory) and quantitative methods (for establishing effectiveness of the intervention) were used in the “Ten-step training plan for NNMs integrating Kemp model and role theory” (Figure 3).

Step 1: Identify learning needs and objectives, priority, and constraint
    Learning needs and objectives
    NNMs are required to have a clear understanding of the differences between the role of front-line nurses and that of management positions. They should actively adjust their work methods based on feedback information to swiftly adapt to the new role, enhance their core competencies, and drive an improvement in the quality of nursing management.
    Priority factors
    Experienced teaching staff is a key priority. Seasoned nurse educators with clinical and management know-how can offer practical insights. A supportive hospital administration is also crucial, allocating resources like funds for materials and external training.
    Constraint factors
    The lack of dedicated rooms or overbooking disrupts training. Time is another issue, as NNMs, burdened with managerial tasks, struggle to find time for training without neglecting work.
Step 2: Select topics and tasks
Design and application of a core competency training program for NNMs based on the Kemp model from role theory perspective.
Step 3: Analyze NNMs' characteristics
Both qualitative and quantitative methods were employed to analyze the characteristics of NNMs. In the qualitative part, the focus was on exploring the difficulties and needs encountered by NNMs during the role transition process. The analysis of interview results yielded four themes: role ambiguity, role conflict, role learning, and role behaviors. For detailed information, please refer to the published article by the research team [20]. In the quantitative part, the “Nurse Manager Competency Assessment Questionnaire” developed by Lv et al. [37] was used to conduct a pretraining assessment of the core competency levels of NNMs. The scores of NNMs in each competency dimension are shown in Supporting Information S1, and they performed poorly in behavioral competence.
Step 4: Predict NNMs' readiness for learning
During the interviews with NNMs, they expounded on the various preparations they had made and the efforts they had exerted during the role-transition phase. The interview results clearly demonstrated the specific manifestations of NNMs in role learning and role behavior. Meanwhile, the questionnaire conducted a comprehensive evaluation of NMs from multiple aspects, including professional competence. The learning preparedness of NNMs can serve as a crucial reference for instructional design.
Step 5: Utilize teaching resources
Training faculty: the hospital hosting the project has a wealth of expert professors with extensive clinical, teaching, and management experience. They excel in clinical practice and possess deep expertise in education and administration. Moreover, the nursing department has forged strong partnerships with experts from multiple hospitals and academic institutions, ensuring high-caliber teaching resources.
Training materials: the hospital's departments and nursing unit are well stocked with teaching aids, including textbooks and case studies, providing comprehensive resources for NNMs. An electronic library with a diverse range of subscription materials is also available to cater to various learning levels and needs.
Training equipment and venues: the hospital is equipped with cutting-edge intelligent teaching facilities, including remote learning systems, VR classrooms, and training centers, offering a convenient and comfortable training environment for NNMs.
Step 6: Provide ancillary services
This service primarily centers on the psychological and emotional well—being of NNMs. The NM of the department holds one—on—one conversations with NNMs (NM of the ward) every week, and the director of the nursing department conducts one—on—one talks with NNMs on a monthly basis. Through these interactions, the concerns of NNMs can be fully heard, and they can receive empathetic feedback from middle— and high—level nursing administrators, which helps to alleviate their role—related stress to a certain extent.
Step 7: clarify the teaching objectives
Through the guidance of core competency training programs, we aim to empower NNMs to adeptly navigate and resolve intricate role—related challenges and foster heightened self—awareness, enabling them to seamlessly integrate into their new roles post—training. Furthermore, we strive to catalyze a substantial enhancement in their leadership effectiveness and core competence.
Step 8: analyze the subject content
The framework of this research curriculum was developed based on the AONL Nurse Leader Core Competencies framework, interview results, and competency assessment levels. This study employed the Brislin translation model to translate the AONL framework into Chinese [38]. Seven experts on nursing management with bilingual literacy in Chinese and English were invited to rate the initial Chinese version of the AONL Nurse Leader Core Competencies framework from the perspectives of language equivalence and cultural relevance using a 4-point Likert scale. The percentages of experts who provided ratings of 3 and 4 were calculated, and their comments were considered in revising the initial Chinese version of the framework. Regarding language equivalence and cultural relevance, the seven experts agreed 100% for all items.
After three one-hour group meetings, the research team established an initial course structure with 29 modules and 38 items. Fifteen experts participated in two rounds of Delphi consultations on the curriculum for NNMs, achieving a 100% response rate. The expert panel's basis of judgment was 0.933 and 0.927, with familiarity with the content of 0.807 and 0.820, respectively. Authority coefficients of experts were 0.870 and 0.873. Coefficients of variation ranged from 0.00 to 0.22 and 0.00 to 0.23, with statistically significant Kendall's coefficients of 0.229 and 0.202. Ultimately, a competency-based curriculum for NNMs was developed, featuring 30 modules and 43 items (Supporting Information S2).
Step 9: implement teaching activities
    Core competency-based training course
    A hospital nursing management training program is established, targeting the competency development of NNMs. The program organized courses within the hospital to train NNMs in the required competencies. After a literature review and expert discussions, the specific implementation strategies for the training courses are formulated as follows: ① Training content: a competency-based training curriculum for NNMs, grounded in the AONL framework, interview results, and competency assessment levels; ② Training methods: the primary method is lecture based, supplemented by scenario simulations, workshops, group discussions, and experience sharing sessions; ③ Training frequency: the program will be conducted once a week, with each session comprising 2-3 courses, spanning over a period of 3 months; ④ Training location: nursing training center, distance learning classroom, and virtual reality classroom; and ⑤ Instructors: experts and scholars from the hospital affiliated with the research institute, other hospitals, and relevant academic institutions, specializing in fields related to the training course content such as hospital management, education, financial management, healthcare policy research, and high-quality development of nursing.
    “One-to-one mentorship”
    Effective mentors guide others through professional exemplary behavior and personal balancing abilities, thereby embodying positive role model characteristics such as teachers, consultants, exemplars, coaches, and confidants [39, 40]. In this study, along with conducting course training, a mentorship program is implemented for NNMs, establishing specific training strategies to enable them to familiarize themselves with the NM role quickly and smoothly transition into their new positions. The “mentorship system” refers to the assignment of a dedicated “one-on-one” mentor to each NNMs during their first year in the position. A mentor pool is established, and mentors are selected based on the following criteria: over 5 years of experience as a senior nurse manager (SNM); exceeding standard values in nursing unit management and quality control; strong interpersonal skills, including the ability to listen and a willingness to help young nursing directors grow; and strong team cohesion and collaboration abilities. Currently, the hospital's mentor pool consists of 15 mentors. The deputy director of the nursing department trains the selected mentors and clarifies their responsibilities: ① Mentors are expected to maintain a good mentor–mentee relationship with the NNMs during the training period, providing support and assistance and openly discussing any difficulties and issues and ② During the training, there will be one on-site exchange and learning session in the ward per week, tailored to the course content of the training program for that week and the individual needs of NNMs, spanning over a period of 3 months. For one-on-one mentor matching, NNMs select a dedicated mentor from the mentor pool based on factors such as specialty alignment, mentor characteristics, and personal personality. For example, a NNM in general surgery would choose a SNM in general surgery from the mentor pool. If there is no corresponding SNM in the specific specialty, a SNM in a related surgical field would be selected.
    Reflective journal
    A reflective journal requires students to critically review their learning process and outcomes in writing, stimulating learning enthusiasm and deepening understanding of acquired knowledge [41, 42]. This model is chosen due to its successful use in nursing [42, 45]. Develop a manual for NNMs on writing reflective journals. This manual consists of six modules: reflection, description, feeling, evaluation, analysis, conclusion, and action plan. Provide training and guidance to NNMs on writing reflective journals, emphasizing the significance of this practice. During the training period, NNMs were required to fill out the reflective journal manual once a month and submit it to the project leader. The project leader will review the students' reflective journals on time and add brief comments at the end of each journal entry.
Step 10: conduct teaching evaluations
  General Information Questionnaire
  Based on a review of relevant literature, a general information questionnaire was developed, incorporating relevant variables according to the research objectives, including gender, age, educational background, department, professional title, and duration of serving as a NM (in months).
  Formative evaluation
      Classroom questioning
      It serves to stimulate the active thinking of NNMs, enabling an instant understanding of their comprehension of the training content. It allows for the timely correction of misconceptions and guides them to delve deeper into the knowledge and skills related to core competence.
      Homework
      It is designed to consolidate what has been learned in the classroom, strengthen the practical application of core competencies by NNMs, cultivate their abilities in autonomous learning and independent problem-solving, and simultaneously offer insights to trainers regarding the learning difficulties encountered.
      Reflective journal
      Each NNM was required to submit four reflective journals, including three periodic reflective diaries written during the training period and one summative reflective diary completed after the final training session.
  Summative evaluation
      Nurse Manager Competency Assessment Questionnaire
      This study utilized the Nurse Manager Competency Assessment Questionnaire developed by Lv et al. [37] to evaluate the competency levels of NNMs 1 month after completing the training. The questionnaire consists of 4 dimensions: professional competence (6 items), ethical competence (5 items), psychological competence (10 items), and behavioral competence (12 items), with a total of 33 items. Each item is scored on a 5-point Likert scale, ranging from “completely disagree” to “completely agree,” with scores of 1–5 respectively. A higher mean score for the items indicates a stronger competency of the NNMs. The Cronbach's α coefficient of this questionnaire is 0.945, and the content validity is 0.960.
      Semistructured interview
      Based on Role Theory, this study formulated a semi-structured interview guide to explore the role adaptation of NNMs 1 month after completing the training. The content is as follows: ① Do you think the competency training has been helpful for your role transition? In which aspects (knowledge, skills, abilities, qualities, etc.) have you seen improvement? ② Compared to the challenges or bottlenecks you faced during the role transition before attending the training program, what needs have this training program fulfilled for you? ③ After the training, has your understanding of the nurse leader role changed? What other abilities do you still want to improve? ④ Please share your future plans for this role and how attending this training program has influenced your career development prospects.

### 2.6. Data Collection

Both interviewers (the first and second authors) are registered nurses with Master's degrees in Nursing and over 2 years of clinical experience and have completed systematic training in qualitative interview techniques to ensure methodological rigor. The interview times were determined autonomously by the participants, and the entire process took place in a private and confidential space within the hospital. There was no prior contact between the researchers and participants to minimize the risk of social bias. Participants were clearly informed about the research background, the scope of data usage, and their right to withdraw at any time. Audio recordings were anonymized and used solely for academic analysis, ensuring privacy and ethical compliance. The researchers dynamically adjusted the depth of the questions based on feedback and concurrently documented nonverbal behaviors (such as body language and emotional fluctuations) to supplement the textual information. Questions were framed in a neutral manner to reduce the risk of leading bias. Data collection continued until theoretical saturation was reached (i.e., no new themes emerged), with each interview lasting for 30–40 min.

Eligible NNMs were invited to participate in a questionnaire survey both before and 1 month after the training. After explaining the purpose of the study and obtaining their consent, an electronic link to the questionnaire was sent via WeChat for them to complete and submit online. Follow-up was conducted for incomplete questionnaires, requesting them to be completed within 24 h. During the training, NNMs were asked to write a reflective diary at the end of each month, and a summary reflective diary after the training, amounting to a total of four reflective diaries to be submitted to the researchers.

### 2.7. Data Analysis

Version 25.0 of the SPSS software (IBM Corp., Armonk, NY, USA) was used in the statistical analysis. *p* values < 0.05 were taken as denoting statistical significance. General information was described using frequency, mean, standard deviation, and percentage. For the comparison of measurement data before and after training, paired-sample *t*-tests were used for data that followed a normal distribution and had homogeneous variances, while nonparametric tests were employed for data that did not meet these criteria.

Within 24 h of each interview, the audio recordings were transcribed verbatim into written text. Two researchers independently reviewed, coded, and classified the same text data. The transcripts were coded in chronological order (N1, N2, N3 … N14, 14 codes in total) and imported into Nvivo 12.0 software for sequential processing. The Colaizzi method was used to inductively analyze and refine the interview data [43, 44]. In cases where discrepancies emerged during analysis, the research team convened for a collaborative discussion and analysis to refine the final themes.

Based on role theory, we combed through and categorized the discourse reflecting role cognition elements in the reflective journals and analyzed them to condense the themes of role cognition embodied in each journal. To ensure the reliability of the thematic analysis of the reflective journals, two instructors independently reviewed and analyzed the content of the journals. For those journals with discrepant evaluations, the entire research team collectively discussed and determined their reflective themes.

### 2.8. Ethical Considerations

The research protocol was approved by the Institutional Review Board of Beijing Tiantan Hospital, Capital Medical University (IRB no. KY2023-232-02) on April 19, 2024. All participants provided written informed consent for both research participation and audio-recorded interviews, with explicit assurance of their unconditional right to withdraw at any time without penalty. To ensure strict confidentiality, all collected data were accessible exclusively to the research team through password-protected systems. Participant anonymity was rigorously maintained by replacing identifiable information with unique numerical codes prior to data analysis. Furthermore, interview transcripts distributed to noninterviewing researchers contained fully deidentified content. The research team confirms no pre-existing administrative relationships or conflicts of interest with any participants throughout the study duration.

## 3. Results

### 3.1. Demographic Characteristics

The study includes 14 NNMs, all of whom are women. Their ages span from 30 to 40, with an average of 36.29 ± 3.27 years. Regarding educational attainment, 2 possess Master's degrees while 12 hold Bachelor's degrees. In terms of professional titles, one is an associate senior nurse, and the remaining 13 are intermediate nurses. Their work experience varies between 7 and 20 years, averaging 13.79 ± 3.79 years. As for their assignments, 3 are in the General Medical Department, 3 in the Outpatient Department, 2 in the Critical Care Unit, 2 in Neurosurgery, 2 in Surgery, 1 in Obstetrics and Pediatrics, and 1 in Neurology. See Supporting Information S3 for details.

### 3.2. Competency of NNMs

The scores for competency among the 14 NNMs before and after training followed a normal distribution. Using paired-sample *t*-tests, the results indicated that the total competency score, as well as scores for professional competence, professional ethics and qualities, psychological competence, and behavioral competence, were all higher after training compared with before. Specifically, the differences in the total competency score and behavioral competence score were statistically significant (*p* < 0.05). See Table 1 for details.

### 3.3. Interview and Reflective Journal Results

Based on role theory, this study ultimately extracted five themes (Table 2) from the semistructured interviews and reflective journals of NNMs. These themes are (1) role awareness awakening and cognitive restructuring, (2) capability adjustment in role practice, (3) role identity and coconstruction of team culture, (4) the tension between role expectations and career development, and (5) role cultivation and continuous professional development. As can be seen from these themes, the role development of NNMs unfolds along a dynamic five-stage trajectory: “awareness awakening—practice adjustment—identity elevation—expectation restructuring—sustained development.”

### 3.4. Role Theory-Driven Themes

#### 3.4.1. Role Awareness Awakening and Cognitive Restructuring

##### 3.4.1.1. Deepening and Expansion of Role Positioning

Through training and work practice, NNMs gradually transform from single-role nursing practitioners to compound managers, redefining their professional identities and the boundaries of their responsibilities. This reflects a deepening process of role positioning, evolving from simplicity to comprehensiveness. As one interviewee stated:

“Initially, as a seasoned nurse, I perceived the nurse manager's role as just an ordinary management position. However, after nearly seven months of work and training, I've realized that this grassroots management role encompasses a wide range of responsibilities, covering people, tasks, and resources in every aspect” (N1).

In addition to the in-depth development of role positioning, the NM position requires a macro perspective and forward-thinking insight to discern nursing trends and chart a goal-oriented path for the team that aligns with the hospital's development. This highlights the expansion and elevation of the NM's role from a local to a macrolevel. As one interviewee commented:

“The training has made me realize that nurse managers are not only the direct commanders of daily nursing work but also important participants in the strategic planning of hospital nursing. Nurse managers need to have a long-term vision, be able to anticipate trends in the nursing field, and formulate clear development goals and implementation paths for the team” (N3).

##### 3.4.1.2. Systematic Construction of Management Thinking

After training, the management thinking of NNMs has undergone a transformation from being singular and one-sided to comprehensive and systematic. Whether it is the all-encompassing management thinking across multiple dimensions such as nursing quality, teaching, and research or the courage to translate macro goals into actionable plans, it reflects the systematic construction of their management thinking in terms of seamless integration and precise implementation.

“The training courses have given me a clearer understanding that in the future, I need to focus on development in areas such as nursing quality, clinical teaching, talent cultivation, team building, professional development, and nursing research” (N4).

“Through the training, I've realized that as a nurse manager, I should be an efficient executor, translating the hospital's overarching direction into departmental objectives and leading the team to achieve these goals” (N6).

Two other interviewees also expressed similar viewpoints, emphasizing that a holistic perspective that transcends departmental self-interest and measures to empower team members have made management thinking more focused on team collaboration and overall effectiveness, further refining the systematic management approach.

“When dealing with relationships with other departments, it's important to have a holistic perspective and avoid departmental self-interest. We shouldn't always approach problems solely from the standpoint of our own department” (N11).

“The strength of a nurse manager alone is limited. By leveraging the strengths and unique talents of each nurse and incorporating them into ward management, we can foster a sense of participation and ownership among the nursing staff, enabling us to collectively manage the ward” (N13).

#### 3.4.2. Capability Adjustment in Role Practice

##### 3.4.2.1. Clinical Decision-Making and Crisis Management Capabilities

Through training during role practice, NNMs continually refine their clinical decision-making and crisis management abilities. They not only hone their professional decision-making skills but also gain a profound understanding of the vital need to assume responsibility in crisis situations, thereby better fulfilling the demands of their managerial role. As the interviewees noted:

“Through problem-solving exercises and decision-making training that simulate real-world work scenarios, I have learned how to apply critical thinking to analyze the root causes of problems, employ various strategies to develop effective solutions, and make reasonable judgments swiftly in complex and ever-changing environments” (N2).

“The meticulously designed courses and case studies in the training program have helped me build a comprehensive knowledge system, enabling me to quickly identify the root causes of problems and devise solutions” (N5).

“When faced with urgent, difficult, dangerous, and heavy work tasks, nurse managers must dare to rise to the challenge and take responsibility” (N12).

##### 3.4.2.2. Cross-Departmental Collaboration and Resource Integration

In their role practice, NNMs are actively exploring ways to enhance their cross-departmental collaboration and resource integration capabilities. They recognize the importance of improving their own abilities in facilitating smooth cross-departmental work and achieving optimal resource allocation so as to better meet the demands of the NM role. As two interviewees stated:

“I plan to enhance my leadership and team collaboration skills by actively participating in leadership training programs, team-building activities, and cross-departmental cooperation projects” (N7).

“Effective communication is the cornerstone of team collaboration. I hope to further improve my communication skills, especially in cross-departmental cooperation and conflict resolution, through additional learning” (N9).

#### 3.4.3. Role Identity and Coconstruction of Team Culture

##### 3.4.3.1. Team Motivation and Potential Activation

During the training process, NNMs have deepened their role identity and recognized their crucial responsibilities in team motivation and potential activation within the context of coconstructing team culture. They are actively exploring effective ways to drive team development and better fulfill their roles as NMs. Two interviewees focused on the leading and supporting roles of NMs in team building, highlighting their key responsibilities in fostering a positive team environment and promoting team growth.

“In the high-pressure environment of nursing work, nurse managers must also excel at emotional management, utilizing effective communication and team-building strategies to create a positive work atmosphere, alleviate team members' stress, and enhance overall cohesion” (N1).

“Nurse managers should enhance their own stress resilience, provide sufficient freedom and space for team development, and lead the team towards growth” (N8).

In addition, two other interviewees demonstrated profound care and high regard for the individual development of team members, embodying a people-oriented management philosophy. This approach helps cultivate a positive, collaborative, and supportive team atmosphere, enabling team members to achieve a unity of personal and team values through mutual support and shared growth.

“Nurse managers should tap into nurses' potential, leverage their strengths, assist them in establishing career development plans, and guide them in achieving set goals” (N10).

“I have clarified my role in promoting team development. For me, enhancing my own resilience, providing the team with ample freedom and space for growth, and guiding their progress are of utmost importance. As for each nurse, I have a responsibility to stimulate their potential and capabilities” (N11).

##### 3.4.3.2. Shaping Cultural Symbols and Collective Memory

During the process of role identification, NNMs gradually come to realize that team culture needs to be vividly manifested and perpetuated through tangible cultural symbols and the collective memories shared by team members. To this end, they actively organize various activities aimed at creating distinctive cultural symbols and accumulating cherished collective memories.

“On the occasion of International Nurses' Day on May 12th, I organized a small gathering for the ward nurses, complete with a communal lunch and small gifts for each nurse. This helped to bridge the gap between us, giving the nurses a sense of belonging and earning their recognition” (N2).

“I encouraged everyone to actively participate in the hospital's sports meet and took care of the logistical support for the participants. We created a commemorative vlog of the event and displayed promotional materials about the sports meet on the notice board in the ward's living area. This enhanced the nurses' sense of honor, participation, and achievement, making them feel the warmth and strength of unity, and ultimately boosting the cohesion of our department” (N13).

#### 3.4.4. The Tension Between Role Expectations and Career Development

##### 3.4.4.1. Shaping Professional Authority and Leadership

As the core figure of the team, NMs possess solid professional knowledge and exquisite skills, enabling them to set professional benchmarks within the team, earn the respect and trust of team members, and guide the team toward improving nursing quality. This underscores the foundational role of professional authority in the role of a NM, as expressed by one interviewee:

“When the nurse manager pays attention to nursing quality, the team will follow suit. Therefore, the professional competence and level of the nurse manager are of utmost importance” (N3).

Similarly, interviewees also recognize that maintaining a continuous learning attitude and keeping pace with industry trends are essential to meeting the growing demands of the position and maintaining professional authority in the field.

“Continuous learning is necessary to constantly update knowledge and skills, better adapting to the evolving needs and changes of the position” (N9).

During the process of quality control and rectification, effective communication enables nurses to embrace the measures, while leadership garners trust and support, motivating team enthusiasm. This demonstrates that enhancing leadership is a necessary condition for NMs to transform professional authority into team influence and drive overall team development.

“In the role of a nurse manager, one needs to navigate and manage interpersonal relationships, meeting the demands of quality control and rectification while also gaining the nurses' trust. This requires enhancing leadership skills” (N14).

##### 3.4.4.2. Phased Reconstruction of Career Planning

The phased reconstruction of career planning during the training process reflects NNMs' proactive response to the role expectations of NMs and demonstrates their initiative and strategic thinking in career development. Both interviewees based their planning on the actual conditions of their wards, establishing time milestones. They first focused on consolidating foundational knowledge to ensure smooth ward operations and then devoted themselves to scientific research, thereby constructing an upward pathway from practice to research for both personal and team development.

“My career plan is to comprehensively master the diseases and nursing knowledge in our ward within one year; in the second year, I will commence scientific research work” (N1).

“Within two years of serving as a nurse manager, I will streamline various processes in the ward. After ensuring stable ward operations, I will drive scientific research among nursing staff” (N10).

Meanwhile, one interviewee also took a macro perspective in their career planning. By engaging in diverse learning methods, they sought to elevate their professional expertise, keep pace with the latest industry trends, and continuously refresh their knowledge framework. This approach embodies notable foresight and systematic thinking, allowing them to accumulate capabilities for long-term development and align with the high-quality standards demanded by their role.

“In the coming years, I am committed to continuously improving my professional skills by reading the latest nursing literature, attending renowned seminars, and pursuing advanced courses” (N4).

#### 3.4.5. Role Cultivation and Continuous Professional Development

##### 3.4.5.1. Reflective Practice and Knowledge Transformation

Reflective practice serves as the core driving force for the growth of NNMs, while knowledge transformation is the crucial step in converting the outcomes of reflective practice into practical management effectiveness. The field of nursing management is far from static; instead, it is rife with dynamic changes and challenges. Only by deeply integrating and closely combining acquired knowledge and skills with actual work can NMs enhance their overall role cultivation and establish a foothold in the complex and ever-changing nursing environment.

“By employing scientific management, I can rationally utilize human resources and establish a sound performance appraisal and distribution system” (N5).

“The time management and stress regulation courses taught me how to plan my time, allocate tasks, and maintain work efficiency, thereby improving my productivity and enabling me to strike a balance between work and personal life” (N8).

“This learning experience has enabled me to understand how to utilize scientific management tools for data collection. By analyzing this data, I can identify quality control issues and precisely pinpoint areas in my ward that require improvement” (N9).

##### 3.4.5.2. Service Optimization and Innovation Leadership

Advancements in medical technology have provided more tools and possibilities for nursing practice. As the core and soul of the nursing team, NMs must possess a forward-looking strategic vision and a pioneering spirit willing to break through conventions. With innovation as the driving force and service optimization as the goal, they should actively promote changes in nursing models and enhance service quality, thereby adding new dimensions to the role cultivation of NMs.

“Against the backdrop of continuous advancements in medical technology and increasingly diverse patient needs, nurse managers must become drivers of innovation in nursing models, leading their teams to explore more efficient and humane nursing service approaches to adapt to the ever-evolving healthcare environment” (N6).

“In the research endeavors of our department, it is noteworthy that our ward leads the nation in the treatment of cerebrovascular diseases. Nursing practice must now strive to catch up and actively engage in innovative research within our field” (N14).

## 4. Discussion

The findings of this study demonstrate that the training program combining the Kemp model and role theory significantly enhanced the core competencies of NNMs and facilitated their role adaptation and role development. This highlights the importance of implementing holistic instructional design and personalized role adaptation in core competency training programs for NNMs. Based on this, hospital administrators should fully recognize the profound significance of this innovative training model for the cultivation and development of NNMs. Taking this as an opportunity, they should continuously improve the NNMs training mechanism by optimizing the training system, establishing training archives, and promoting the application and transformation of training outcomes, aiming to cultivate high-quality and professional nursing management talents. This approach is also in line with the concept advocated by the ICN, which is to establish a “leadership pipeline” [46] to meet the needs of NMs during the transition period. In additionally, it contributes to advancing the goal set forth in the China's “14th Five-Year Plan for Nursing Development,” which is to establish a competency-based training system to bridge the gap between clinical and management roles [22].

The Kemp model ensures that training precisely meets actual needs, while role theory enhances NNMs' understanding of their professional roles [47]. The Kemp model emphasizes a learner-centered approach, conducting thorough and detailed needs analyses of trainees before training. For NNMs, this model accurately identifies specific challenges and knowledge/skill gaps they encounter during the transition from clinical nursing to managerial positions. Based on these precise insights, training content can be tailored to ensure that every activity closely aligns with the capabilities NNMs urgently need to enhance in their practical work. Role theory, on the other hand, focuses on individuals' comprehension and identification with the roles they play. When NNMs participate in targeted training designed using the Kemp model, role theory guides them in deeply considering the unique responsibilities, scope of authority, and behavioral norms associated with nursing management roles. Through interactive teaching methods such as case studies and role-playing during training, NNMs can personally experience the role requirements in various management scenarios, thereby continuously deepening their understanding of their professional roles. The synergy between this precision-oriented training based on needs and the process of deepening role cognition enables NNMs to clearly recognize the core competencies required for their new roles. This, in turn, facilitates targeted learning and improvement, laying a solid foundation for their role adaptation.

The study findings reveal a significant improvement in the behavioral competence dimension, indicating that NNMs have successfully internalized the cognitive framework required for nursing management roles, mastered management skills, and applied them in practice. For instance, areas such as “staff motivation and development” and “team building” not only demand technical skills but also rely on emotional intelligence and a clear understanding of the leader's role, which can be cultivated and reinforced through role identification [48]. The synergy between the Kemp model and role theory in the training program facilitated NNMs' rapid adaptation to new work rhythms and environments, enabling a successful transition from clinical nursing to nursing management. Yet, post-training enhancements in professional, ethics, and psychological competence were limited. This may stem from these abilities' inherent nature. Professional competence is usually honed in clinical roles and is a management prerequisite [17]. Ethics and qualities are judged by moral reputation built over time [49]. Psychological capabilities, with their indirect and long-term impact on management, need repeated role internalization and show changes only after prolonged intervention [37, 48]. Therefore, future research could design a “3–5-year leadership incubation program” with a phased role advancement pathway, providing tailored training and practical opportunities for NNMs at each stage to continuously unlock their management potential.

The themes emerging from the results reveal a five-stage dynamic trajectory in NNMs' role development: “awareness awakening—practice adjustment—identity elevation—expectation restructuring—sustained development.” At the initial awareness awakening stage, NNMs undergo a pivotal transformation from mere nursing practitioners to versatile managers through training and practice, redefining their professional identity and responsibilities—a core indicator of role awareness awakening. Role theory also underscores individuals' behavioral adjustments and cognitive development in role-playing [50]. Training enables NNMs to perceive nursing trends from a macro perspective and plan long-term team strategies, achieving a leap in role orientation from local to macro levels. This aligns with the of findings Du et al. [51], where management practice training based on goal-setting concepts helped NNMs better navigate role transitions and establish clear self-identity. It also echoes the research of Yin et al. [27], indicating that transformative leadership training effectively leverages NNMs' roles in self-improvement and management decision-making.

As training progresses, NNMs enter the role practice adjustment stage. Here, they continually refine their clinical decision-making and crisis management capabilities through training, engaging in problem-solving and decision-making exercises that simulate real-world scenarios. This helps them employ critical thinking to analyze problem roots and devise effective solutions, enabling rapid and rational judgments in complex environments. For instance, the reflections of N2, N5, and N12 demonstrate their proactive adjustment of clinical decision-making and crisis management skills in practice. This is consistent with Xue et al.'s [52] findings that immersive scenarios aid NNMs in embodying their roles and making informed decisions in emergencies, accelerating their transition to management roles and reducing role conflicts.

As role practice deepens, NNMs gradually enter the stage of identity elevation, where training serves as a vital force driving the continuous deepening of their role identity. Role theory offers a robust theoretical explanation for NNMs' role behaviors and contributions in team culture building [29]. As pivotal figures in their teams, NNMs play crucial roles as leaders and facilitators in shaping team culture [53]. By fostering a positive and uplifting team culture, they guide team members under shared values and behavioral norms, cultivating strong cohesion and unity. This team culture not only sparks team members' enthusiasm and creativity but also provides solid cultural support for the team's steady development, further deepening NNMs' role identity and broadening the scope and depth of their role perception.

The expectation restructuring stage focuses on the tension between the NM's role expectations and career development, highlighting the significance of reconstructing professional authority, leadership cultivation, and career planning in stages. Professional authority serves as the cornerstone of the NM's role, with their expertise influencing the team's focus on nursing quality [54], and continuous learning being essential for maintaining this authority. Leadership enhancement is a prerequisite for translating professional authority into team influence; in quality control improvements, effective communication and leadership can galvanize team enthusiasm, aligning with Yin et al.'s [27] emphasis on leadership's role in enhancing nursing team effectiveness. Regarding career planning, NNMs demonstrate proactivity and strategic thinking, not only establishing a progression path from practice to research based on ward realities but also adopting a macro perspective to align with the high-caliber requirements of their roles. This provides diverse insights for head nurses' career development, effectively alleviating the tension between role expectations and career growth.

As the training draws to a close, NNMs enter the final stage–sustained development. At this stage, the concepts, knowledge, and skills imparted by the training have been deeply integrated into NNMs' role perception, becoming a powerful driving force for their continuous growth. Reflection and innovation serve as the core pillars of NNMs' sustained development. They not only enhance the precision of nursing management but also strengthen NNMs' ability to navigate complex nursing environments, providing ongoing impetus for their role refinement. A previous study reported [55] that incorporating feedback-based mentorship and coaching support into NNMs' development programs yields better outcomes. This has been further validated in our training design, where reflective journals and one-on-one mentorship are integrated throughout the program, complemented by organizational support from the nursing director, addressing gaps identified in prior research [55]. Furthermore, NMs occupy a pivotal position at the intersection of nursing teams and senior management, acting as an indispensable bridge for implementing and fostering innovative practices within the nursing team [56]. By actively promoting an innovative culture, NMs can effectively guide team members in adopting new nursing practices, thereby significantly enhancing patient care quality [57]. As demonstrated by the reflective exercises and interviews in this study, NNMs are wholeheartedly committed to becoming proactive drivers of nursing model innovation.

To sum up, the training program integrating the Kemp model and role theory has shown certain effectiveness in enhancing the core competencies of NNMs and facilitating their role adaptation and development. The five stages of NNMs' role evolution vividly illustrate their transformation from individuals to team leaders. For the future, it is recommended to establish a long-term training mechanism. First, consistently optimize the training programs based on the Kemp model and role theory. Tailor and update the training content according to the needs of NNMs at different developmental stages to ensure its relevance and timeliness. Second, provide NNMs with a more diverse array of continuous learning resources, such as online learning platforms, academic exchange events, and practical case repositories, to meet their learning needs in various contexts. This will enable NNMs to fully play a leading role and drive the nursing industry to achieve new breakthroughs in management, service, and other areas.

## 5. Limitations

From a methodological perspective, while the effective integration of the Kemp model and role theory enhances the scientific rigor of competency training for NNMs, the study inevitably has some limitations. First, as a pre–post quasiexperimental pilot study, this research lacks random grouping and parallel control groups, which restricts the ability to infer causal relationships. Moreover, the training was conducted solely in a single tertiary hospital with a limited sample size. Although it serves a certain purpose in feasibility testing, the small sample size constrains the generalizability of the study findings to a broader population. Future research should strive to carry out large-scale, multicenter randomized controlled trials or longitudinal studies to investigate the effectiveness of this training model and further explore the phased characteristics of capability internalization and the context-dependent mechanisms. Second, the participants in this study did not cover all hospital departments, particularly lacking subjects from specialized areas such as operating rooms and emergency departments. To enhance the applicability of the research results and the training program, future studies will expand the range of participant sources.

## 6. Conclusion

This study demonstrates that the training program integrating the Kemp model and role theory can effectively address the difficulties and challenges NNMs face during role transition, enhance their core competencies, and facilitate their role adaptation. The training evaluation results in this study have been summarized into five key themes: role awareness awakening and cognitive restructuring, capability adjustment in role practice, role identity and coconstruction of team culture, the tension between role expectations and career development, and role cultivation and continuous professional development. In addition, a preliminary exploration has been made into the role development path of NNMs during the training process: awareness awakening—practice adjustment—identity elevation—expectation restructuring—sustained development. Given the complexity of nursing management roles and the continuous evolution of healthcare policies, there is currently a scarcity of applied research aimed at promoting the role transition of NNMs. The findings of this study confirm the promoting effect of the structured training mechanism (the integrated framework of Kemp model and role theory) on core competencies, role adaptation, and development. The efficiency of NNMs' role adaptation directly impacts nursing quality, team stability, and medical safety indicators. The training model validated in this study can provide reusable strategies for healthcare institutions to optimize support systems during the managerial transition period.

## 7. Implications for Nursing Management

This study's findings confirm the application value of the integrated Kemp model–role theory framework in the role transition of NNMs, offering valuable practical guidance for formulating and optimizing strategies to enhance NNMs' leadership. These strategies are applicable to both developing and developed countries. Previous policies often overlooked NNMs' strategic leadership, transformational leadership, and the challenges during role transitions, making it difficult for them to fulfill their roles effectively. It is worth considering integrating the Kemp model, which emphasizes flexibility, continuous feedback, and learner characteristics, along with role theory, which highlights role perception and needs analysis, into national or regional nursing leadership curricula. Policymakers can adapt to the evolving healthcare environment, heed NNMs' voices, standardize training quality, and align these initiatives with the global trend of competency-based nursing education. This will contribute to cultivating a more resilient nursing management workforce.

## Figures and Tables

**Table 1:** Changes in competency of new nurse managers (scores, $\bar{x}\pm s$).

Time	Total competence	Professional competence	Professional ethics and qualities	Psychological competence	Behavioral competence
Before training	134.93 ± 11.81	24.64 ± 2.68	22.36 ± 1.65	41.29 ± 3.56	46.64 ± 5.47
After training	146.79 ± 13.92	26.14 ± 2.48	23.00 ± 2.11	44.71 ± 4.32	52.93 ± 5.72
*t*-value	−2.198	−1.412	−0.898	−2.130	−2.570
*p* value	0.047	0.182	0.385	0.053	0.023

**Table 2:** Mapping of deductive themes.

Themes	Subthemes
Role awareness awakening and cognitive restructuring	① Deepening and expansion of role positioning; ② Systematic construction of management thinking

Capability adjustment in role practice	①Clinical decision-making and crisis management capabilities; ② Cross-departmental collaboration and resource integration

Role identity and coconstruction of team culture	① Team motivation and potential activation; ② Shaping cultural symbols and collective memory

The tension between role expectations and career development	① Shaping professional authority and leadership; ② Phased reconstruction of career planning

Role cultivation and continuous professional development	① Reflective practice and knowledge transformation; ② Service optimization and innovation leadership

## Data Availability

The data that support the findings of this study are available on request from the corresponding author. The data are not publicly available due to privacy or ethical restrictions.

## Supporting Information

Supporting Information 1 Supporting Information S1: Scores for each dimension of the competence assessment questionnaire for new nurse managers.

Supporting Information 2 Supporting Information S2: The competency-based curriculum for new nurse managers.

Supporting Information 3 Supporting Information S3: General information of new nurse managers.
