# Supplementary material for: Design and Application of a Core Competency Training Program for New Nurse Managers Based on the Kemp Model From Role Theory Perspective: A Pilot Study
Source: J Nurs Manag. 2025 Sep 8;2025:2702060. doi: 10.1155/jonm/2702060 (PMC12436007; doi:10.1155/jonm/2702060)
Supplement: Supporting Information 1 — Supporting Information S1: Scores for each dimension of the competence assessment questionnaire for new nurse managers. [file 2702060.f1.docx]

Supplementary Material S1 Scores for each dimension of the competence assessment questionnaire for new nurse managers

|  | Professional competence | Professional ethics and qualities | Psychological competence | Behavioral competence |
| --- | --- | --- | --- | --- |
| Dimension score | 24.64 | 22.36 | 41.29 | 46.64 |
| Dimension total score | 30.00 | 25.00 | 50.00 | 60.00 |
