# Supplementary material for: Design and Application of a Core Competency Training Program for New Nurse Managers Based on the Kemp Model From Role Theory Perspective: A Pilot Study
Source: J Nurs Manag. 2025 Sep 8;2025:2702060. doi: 10.1155/jonm/2702060 (PMC12436007; doi:10.1155/jonm/2702060)
Supplement: Supporting Information 2 — Supporting Information S2: The competency-based curriculum for new nurse managers. [file 2702060.f2.docx]

**Supplementary Material S2 The competency-based curriculum for new nurse managers**

| **Course classification and titles** | **Course duration** | **Training**  **method** |
| --- | --- | --- |
| 1 Communication and Relationship Building |  |  |
| 1.1 Effective Communication |  |  |
| 1.1.1 Non violent communication between medical staff, nurses, and patients | 2 hours | Lecture; scenario simulations; experience sharing |
| 1.2 Relationship Management |  |  |
| 1.2.1 Establishment and maintenance of relationships with social organizations, media, enterprises, and other groups | 1 hour | Lecture; experience sharing |
| 1.3 Influencing Behaviors |  |  |
| 1.3.1 Analysis, handling, and prevention of common nursing complaints | 2 hours | Experience sharing; group discussions |
| 2 Health Care Environment and Clinical Principles |  |  |
| 2.1 Nursing Practice and Application |  |  |
| 2.1.1 Development and implementation of nursing care plans | 1 hour | Lecture |
| 2.2 Economics and Policy |  |  |
| 2.2.1 Interpretation of medical insurance payment policies | 1 hour | Lecture; experience sharing |
| 2.2.2 The current status of basic healthcare in China | 1 hour | Lecture |
| 2.2.3 Nursing fee item management and practices | 1 hour | Lecture; experience sharing |
| 2.3 Regulation |  |  |
| 2.3.1 Interpretation of nursing laws and regulations | 1 hour | Lecture; group discussions |
| 2.4 Evidence-Based Practice |  |  |
| 2.4.1 Cultivation of evidence-based nursing thinking and translation of evidence | 1 hour | Lecture |
| 2.5 Patient Safety |  |  |
| 2.5.1 Continuous improvement of nursing quality | 3 hours | Lecture; scenario simulations; workshops |
| 2.5.2 Nursing quality control indicator management and monitoring |  |  |
| 2.5.3 Establishment and practice of safety culture-based on clinical nursing practice |  |  |
| 3 Leadership |  |  |
| 3.1 Systems and Complex Adaptive Thinking |  |  |
| 3.1.1 Response to complex problem in nursing management | 1 hour | Experience sharing; group discussions |
| 3.1.2 Cultivation of systematic thinking | 1 hour | Lecture; experience sharing |
| 3.2 Change management |  |  |
| 3.2.1 Change method training and cultivation of change thinking | 1 hour | Lecture; experience sharing |
| 3.2.2 Lean thinking and management | 1 hour | Lecture; experience sharing |
| 3.2.3 Introduction to innovative nursing management models | 1 hour | Lecture; experience sharing; group discussions |
| 3.3 Diversity, Belonging and Inclusion |  |  |
| 3.3.1 Team magnetic culture construction | 1 hour | Lecture; experience sharing |
| 3.4 Decision-Making |  |  |
| 3.4.1 How to make the right decision | 1 hour | Experience sharing; scenario simulations |
| 3.4.2 Art of empowerment | 1 hour | Lecture; experience sharing |
| 3.5 Transformation and Innovation |  |  |
| 3.5.1 Construction of innovative culture in departments | 1 hour | Experience sharing; group discussions |
| 4 Professionalism |  |  |
| 4.1 Profession Accountability |  |  |
| 4.1.1 Sharing of management practices and experiences of excellent nurse leaders | 1 hour | Experience sharing |
| 4.2 Professional Accountability |  |  |
| 4.2.1 The thinking, abilities, and qualities that nurse managers should possess | 1 hour | Lecture |
| 4.3 Advocacy |  |  |
| 4.3.1 Opportunities for nursing development under existing policies | 1 hour | Lecture |
| 4.4 Health Equity and Social Determinates of Health |  |  |
| 4.4.1 Lifecycle health management | 1 hour | Lecture |
| 4.5 Governance |  |  |
| 4.5.1 Development of nurse leader’ s organizational and coordination abilities | 1 hour | Experience sharing; group discussions |
| 5 Business Skills and Principles |  |  |
| 5.1 Professional knowledge and clinical nursing skills |  |  |
| 5.1.1 Development of specialized nursing | 2 hours | Lecture; workshops |
| 5.1.2 Specialized nurse training |  |  |
| 5.2 Scientific Research Management |  |  |
| 5.2.1 Cultivation of nursing scientific research thinking and ability | 1 hour | Lecture; experience sharing |
| 5.2.2 Implementation and quality control of nursing scientific research | 1 hour | Lecture; experience sharing |
| 5.3 Teaching management |  |  |
| 5.3.1 Planning, implementation, and evaluation of clinical nursing teaching | 1 hour | Lecture; experience sharing; scenario simulations |
| 5.4 Financial Management |  |  |
| 5.4.1 Department consumables and material management | 1 hour | Lecture; experience sharing |
| 5.4.2 Performance management | 1 hour | Lecture; experience sharing |
| 5.5 Strategic Management |  |  |
| 5.5.1 Nursing operation management | 1 hour | Lecture; experience sharing |
| 5.5.2 The formulation, application, and evaluation of nursing development strategies | 1 hour | Experience sharing; group discussions |
| 5.6 Human Resource Management |  |  |
| 5.6.1 Evaluation of nursing workload and allocation of human resources | 1 hour | Lecture; experience sharing |
| 6 Leader Within |  |  |
| 6.1 Reflective practice |  |  |
| 6.1.1 Summary and reflection on nursing work | 1 hour | Experience sharing |
| 6.2 Foundational Thinking |  |  |
| 6.2.1 Cultivation of management thinking | 2 hours | Lecture; workshops |
| 6.2.2 Cultivation of statistical thinking |  |  |
| 6.2.3 Cultivation of information thinking |  |  |
| 6.3 Stress and Emotion Management |  |  |
| 6.3.1 Stress and emotion management | 1 hour | Experience sharing; scenario simulations |
| 6.4 Time Management |  |  |
| 6.4.1 Reasonable time planning | 1 hour | Lecture; experience sharing |
| 6.5 Career Development Sub Domains |  |  |
| 6.5.1 Nursing career development planning | 1 hour | Lecture; experience sharing |
| 6.6 Personal and Professional Accountability |  |  |
