# Supplementary material for: Design and Application of a Core Competency Training Program for New Nurse Managers Based on the Kemp Model From Role Theory Perspective: A Pilot Study
Source: J Nurs Manag. 2025 Sep 8;2025:2702060. doi: 10.1155/jonm/2702060 (PMC12436007; doi:10.1155/jonm/2702060)
Supplement: Supporting Information 3 — Supporting Information S3: General information of new nurse managers. [file 2702060.f3.docx]

**Supplementary Material S3 General information of new nurse managers（n=14)**

| ID | Age（years） | Degree | Department | professional | work experience（years） | Length of tenure（months） |
| --- | --- | --- | --- | --- | --- | --- |
| N1 | 38 | undergraduate | ICU | medium-grade | 14 | 3 |
| N2 | 32 | postgraduate | ICU | middle-rank | 7 | 3 |
| N3 | 36 | undergraduate | Outpatient | middle-rank | 16 | 20 |
| N4 | 39 | undergraduate | Gynecology | middle-rank | 20 | 3 |
| N5 | 36 | undergraduate | International Medical Services | middle-rank | 16 | 20 |
| N6 | 32 | undergraduate | International Medical Services | middle-rank | 10 | 3 |
| N7 | 40 | undergraduate | Outpatient | middle-rank | 18 | 3 |
| N8 | 40 | undergraduate | International Medical Services | middle-rank | 16 | 20 |
| N9 | 30 | undergraduate | Outpatient | middle-rank | 10 | 3 |
| N10 | 38 | postgraduate | Internal department | Senior Professional | 11 | 20 |
| N11 | 35 | undergraduate | Surgical department | middle-rank | 14 | 3 |
| N12 | 39 | undergraduate | Surgical department | middle-rank | 16 | 3 |
| N13 | 39 | undergraduate | Surgical department | middle-rank | 16 | 20 |
| N14 | 34 | undergraduate | Surgical department | middle-rank | 9 | 3 |
